# Supplementary material for: Molecular Iodine Supplement Prevents Streptozotocin-Induced Pancreatic Alterations in Mice
Source: Nutrients. 2022 Feb 8;14(3):715. doi: 10.3390/nu14030715 (PMC8840345; doi:10.3390/nu14030715)
Supplement: Supplementary file 1 [file nutrients-14-00715-s001.zip › nutrients-1469969-supplementary.pdf]

Supplementary Table S1. Antibodies used for immunohistochemistry or western blot assays.

Antibody	Dilution	Catalogue #	Manufacturer
Rabbit monoclonal anti-CD34	1 : 1000	ab182981	Abcam
Mouse monoclonal anti-VEGF	1 : 2500	sc-65617	Santa Cruz Biotechnology
Mouse monoclonal anti-insulin B	1 : 500	sc-377071	Santa Cruz Biotechnology
Mouse monoclonal anti-glucagon	1 : 500	sc-514592	Santa Cruz Biotechnology
Mouse monoclonal anti-GFAP	1:500	sc-33673	Santa Cruz Biotechnology
Mouse monoclonal Anti-αSMA	1:500	sc-32251	Santa Cruz Biotechnology
Rabbit polyclonal anti-TNFα	1 : 2500	3707	Cell Signaling Technology
Rabbit monoclonal anti-IL10	1 : 2500	12163	Cell Signaling Technology
Mouse monoclonal anti-TGFβ1	1 : 2500	sc-130348	Santa Cruz Biotechnology
Mouse monoclonal anti-CD8a	1 : 1000	sc-7970	Santa Cruz Biotechnology
Mouse monoclonal anti-iNOS	1 : 200	sc-7271	Santa Cruz Biotechnology
Mouse monoclonal anti-Nfr2	1 : 250	sc-365949	Santa Cruz Biotechnology
Rabbit monoclonal anti-PPARγ	1 : 2500	sc-7196	Santa Cruz Biotechnology
Mouse monoclonal anti-PPARγ phospho ser 112	1 : 2500	MAB3632	Millipore
Mouse monoclonal anti-β Actin	1 : 5000	sc-47778	Santa Cruz Biotechnology
Horse anti mouse IgG biotinylated	1:2000	BA-1000	Vector Laboratories
Goat anti rabbit IgG biotinylated	1:250	BA-2000	Vector Laboratories
Mouse IgGκ BP-HRP	1:10000	sc-516102	Santa Cruz Biotechnology
Goat anti rabbit-HRP	1:5000	sc-2004	Santa Cruz Biotechnology

VEGF, vascular endothelial growth factor, GFAP, glial fibrillary acidic protein; αSMA, smooth muscle actin type alpha; TNFα, tumoral necrosis factor type alpha; IL10, interleukin 10; TGFβ1, transforming growth factor beta; iNOS, inducible nitric oxide synthase; PPARγ, peroxisome proliferator-activated receptor type gamma.

Supplementary Table S2. Primer used for different gene amplification

Gene	Reference	Primer sequence	bp
Catalase	NM_009804.2	FW: 5’-TCCGGGATCTTTTTAACGCCATTG-3’<br>RV: 5’-TCGAGCACGGTAGGGACAGTTCAC-3’	362
Sod1	NM_011434.2	FW: 5’-TGGCCAATGTGTCCATTGAA-3’<br>RV: 5’-TACTGCGCAATCCCAATCAC-3’	181

bp, base pair; Sod1, superoxide dismutase type 1.
